# Post-millennial trends of socioeconomic inequalities in chronic illness among adults in Germany

**DOI:** 10.1186/s13104-018-3299-4

**Published:** 2018-03-27

**Authors:** Jens Hoebel, Benjamin Kuntz, Irene Moor, Lars Eric Kroll, Thomas Lampert

**Affiliations:** 1 Unit of Social Determinants of Health, Department of Epidemiology and Health Monitoring, Robert Koch Institute, General-Pape-Straße 62-66, 12101 Berlin, Germany; 2 Institute of Medical Sociology, Martin Luther University Halle-Wittenberg, Magdeburger Straße 8, 06112 Halle, Germany

**Keywords:** Social determinants of health, Chronic conditions, Health inequalities, Socioeconomic inequalities in health, Health monitoring

## Abstract

**Objective:**

Time trends in health inequalities have scarcely been studied in Germany as only few national data have been available. In this paper, we explore trends in socioeconomic inequalities in the prevalence of chronic illness using Germany-wide data from four cross-sectional health surveys conducted between 2003 and 2012 (n = 54,197; ages 25–69 years). We thereby expand a prior analysis on post-millennial inequality trends in behavioural risk factors by turning the focus to chronic illness as the outcome measure. The regression-based slope index of inequality (SII) and relative index of inequality (RII) were calculated to estimate the extent of absolute and relative socioeconomic inequalities in chronic illness, respectively.

**Results:**

The results for men revealed a significant increase in the extent of socioeconomic inequalities in chronic illness between 2003 and 2012 on both the absolute and relative scales (SII_2003_ = 0.06, SII_2012_ = 0.17, *p*-trend = 0.013; RII_2003_ = 1.18, RII_2012_ = 1.57, *p*-trend = 0.013). In women, similar increases in socioeconomic inequalities in chronic illness were found (SII_2003_ = 0.05, SII_2012_ = 0.14, *p*-trend = 0.022; RII_2003_ = 1.14, RII_2012_ = 1.40, *p*-trend = 0.021). Whereas in men this trend was driven by an increasing prevalence of chronic illness in the low socioeconomic group, the trend in women was predominantly the result of a declining prevalence in the high socioeconomic group.

## Introduction

Over the past decades, public health research has paid increasing attention to the social determinants of health [1–4]. A wide range of studies consistently show that people with lower socioeconomic status (SES) experience poorer health, have increased risk of chronic illness, and die at younger ages than those with higher SES [5–9]. In recent years, a growing number of studies from many European countries have investigated how the health gap between lower and higher SES groups has developed over time, that is, whether the gap has largely remained unchanged over decades or whether it narrowed or widened during some periods [10–17]. In Germany, however, trends in health inequalities have been investigated less often as there were relatively few national data containing both SES and health variables, to establish time series of some length [18–20].

In the early 2000s, Germany started to establish a national health monitoring system administered by the Robert Koch Institute (RKI) on behalf of the German Federal Ministry of Health [21, 22]. Among different health interview and examination surveys, repeated cross-sectional health interview surveys are carried out among the general adult population of Germany to provide data on time trends in population health [23–25]. In two previously published articles, we reported results from a time-trend analysis based on these data. Our findings indicated that social inequalities in tobacco smoking and leisure-time physical inactivity have persisted and even widened among adults in Germany since the early 2000s [26, 27]. In the present paper, we expand this analysis by turning the focus from behavioural risk factors to chronic illness as a health outcome indicator. We explore trends in socioeconomic inequalities in chronic illness among the general adult population of Germany using the same survey data and analytical methods as in the two previously published articles.

## Main text

### Methods

The data used in the analysis were derived from four cross-sectional telephone health surveys among adults living in private households across Germany [22, 23]. The first Germany-wide telephone health survey was conducted in 2003 and was continued in the ‘German Health Update’ (GEDA) surveys in 2009, 2010, and 2012 [23]. Each survey was based on a two-stage sampling procedure. In the first stage, random samples of telephone numbers were generated using random digit dialling. In the second stage, one adult member of each contacted household was randomly selected for interview. Sample sizes of participants aged 25–69 years were n = 6890 in 2003, n = 16,418 in 2009, n = 17,145 in 2010, and n = 13,744 in 2012. Data were collected using standardised computer-assisted telephone interviewing in each of the surveys. Further information on the survey design, contents, response rates, and sample characteristics can be found in the study descriptions [23, 28] and in the two previously published articles presenting time-trend analyses based on these data [26, 27].

In each of the four surveys, chronic illness was assessed by asking all participants the following yes/no question, “Do you have one or more long-lasting chronic illnesses? Chronic illnesses are long-standing diseases requiring constant treatment and monitoring, for example, diabetes or heart diseases”. Participants’ SES was determined using a composite index developed for all surveys conducted by the Robert Koch Institute as components of the German national health monitoring system. The index is an additive index based on information about participants’ educational attainment (school and professional education), occupational position, and net equivalent income. Details on the index and methods used in its construction are described elsewhere [29, 30].

In the statistical analysis, we calculated prevalence rates for chronic illness by SES and sex. We computed predictive margins [31] from logistic regression models to predict age-standardised prevalence rates according to SES, sex, and survey year. Changes in the extent of socioeconomic inequalities in chronic illness were examined by calculating the slope index of inequality (SII) and relative index of inequality (RII) for each survey year [32, 33]. The SII and RII are regression-based summary measures that take into account the entire distribution of a socioeconomic variable as well as the size of socioeconomic groups [33, 34]. The indices complement each other in that the SII quantifies the magnitude of absolute health inequality whereas the RII indicates the magnitude of relative health inequality. Whereas the SII can be interpreted as the age-adjusted prevalence difference between people with the lowest and those with the highest SES, the RII represents the age-adjusted prevalence ratio between these groups. Particularly in time-trend analysis, selective use of exclusively absolute or relative measures of health inequality can lead to biased assessment of increasing or decreasing health inequality over time, which is why it is recommended to consider both measures whenever possible [34, 35].

We used generalised linear regression models for binomial data, with an identity link function (linear probability model) to compute the SII and a logarithmic link function (log-binomial model) to calculate the RII. Changes in the SII and RII over time were analysed by adding an interaction term between SES and survey year to the models while adjusting for age, age × year, and the main effects of SES and year. Weighting factors were used to account for unequal sampling probabilities and to adjust the distribution of each sample by sex, age, education, and region to match the official population statistics for Germany. Analyses were performed using Stata 14.1 (StataCorp LP, College Station, TX, USA) survey data commands.

### Results

**Fig. 1:**
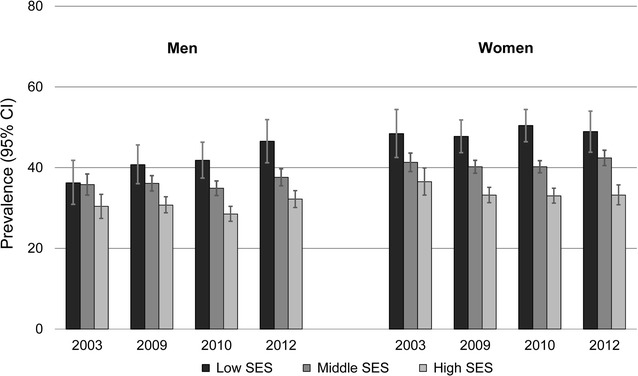
Prevalence of chronic illness among men and women aged 25–69 years in Germany, by socioeconomic status (SES) and year

**Fig. 2:**
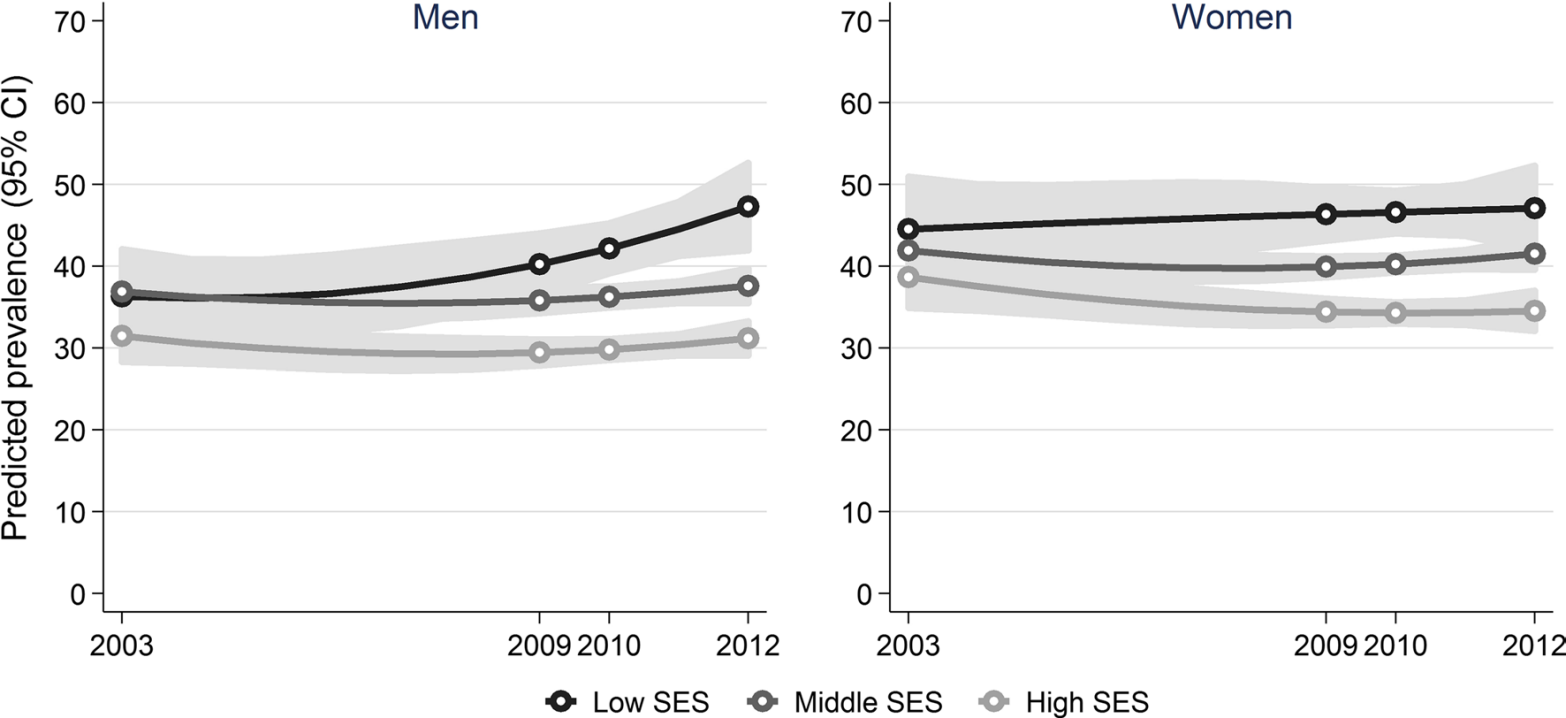
Predicted age-standardised prevalence of chronic illness among men and women aged 25–69 years in Germany, by socioeconomic status (SES) and year (age-standardised to the revised European Standard Population 2013)

Across the study period, the crude prevalence of chronic illness varied between 34.5 and 37.6% among men and between 40.1 and 41.5% among women. As shown in Fig. 1, chronic illness was significantly more prevalent (*p *< 0.001) in lower than in higher SES groups during each survey year and in both sexes, except for men in 2003. Among men in 2003, the crude prevalence differences by SES were not statistically significant either at the 5 or 10% level. Figure 2 shows predictive margins representing age-standardised prevalence rates for chronic illness as predicted by logistic regression. Among men, the results revealed a significant increase in the prevalence of chronic illness in the low SES group from 2003 to 2012 (*p *= 0.009); in the middle and high SES groups, the prevalence was not found to have changed significantly across this period. Among women, the prevalence of chronic illness declined in the high SES group (significant at the 10% level with *p *= 0.063); no significant trend was found in the middle and low SES groups at either the 5 or 10% level.

Table 1 presents the summary measures of absolute and relative inequalities in chronic illness. The result for men revealed a significant increase in socioeconomic inequalities in chronic illness between 2003 and 2012 on both the absolute and relative scales. For women, the results also showed significant increases in absolute and relative inequalities during the study period, although the coefficients tended to fall slightly in the final survey year.

**Table 1:** Trends in absolute and relative socioeconomic inequalities in chronic illness among men and women aged 25–69 years in Germany

	2003	2009	2010	2012	*p*-trend
Men					
SII (95% CI)^a^	0.06 (− 0.01 to 0.13)	0.11 (0.06 to 0.16)	0.15 (0.10 to 0.20)	0.17 (0.11 to 0.22)	0.013
RII (95% CI)^a^	1.18 (0.98 to 1.41)	1.31 (1.15 to 1.51)	1.49 (1.30 to 1.70)	1.57 (1.37 to 1.80)	0.013
Women					
SII (95% CI)^a^	0.05 (− 0.01 to 0.12)	0.13 (0.09 to 0.18)	0.16 (0.12 to 0.20)	0.14 (0.08 to 0.20)	0.022
RII (95% CI)^a^	1.14 (0.97 to 1.34)	1.42 (1.27 to 1.58)	1.47 (1.32 to 1.64)	1.40 (1.23 to 1.59)	0.021

*SII* slope index of inequality, *RII* relative index of inequality, *CI* confidence interval

^a^Adjusted for age

### Discussion

The analysis presented in this paper is the first to investigate post-millennial trends in the extent of socioeconomic inequalities in chronic illness among the general adult population of Germany. The results suggest that both absolute and relative inequalities in chronic illness evolved and widened during the period between 2003 and 2012. Whereas in men this trend was driven by an increasing prevalence of chronic illness in the low socioeconomic group, the trend in women was predominantly owing to a declining prevalence in the high socioeconomic group.

The health outcome considered in the analysis was chronic illness measured by a single question on self-reported chronic morbidity, as is often used in general health surveys. From the literature, it is known that most widespread chronic conditions, such as diabetes, cardiovascular disease, stroke, chronic back pain, chronic bronchitis or depression, are associated with lower SES [36–41]. An exception, however, are allergies, which are generally found to be associated with higher SES [41, 42]. Against this background, it should be considered that the socioeconomic gradient in conditions such as diabetes, chronic back pain or depression might actually be steeper than estimated in our analysis of chronic illness because allergies, which are generally included in the generic definition of chronic illness, may have attenuated the gradient.

Previous studies on trends in socioeconomic inequalities in chronic illness have shown mixed results. Two Scandinavian studies analysed survey data from the mid-1980s and mid-1990s and found stable or slightly decreasing educational inequalities in (limiting) long-standing illness over time [17, 43]. Whereas studies on post-millennial trends in socioeconomic inequalities in chronic illness are generally scarce, findings on other health outcomes exist. A large study based on pooled data from 17 European countries showed that between 1990 and 2010, absolute inequalities in self-rated general health were mostly constant whereas relative inequalities increased [13]. Another analysis of large European data sets revealed that absolute and relative inequalities in functional limitations among older people increased between 2002 and 2014 [44]. Inequalities in single widespread diseases, such as diabetes [15], myocardial infarction or stroke [16, 45, 46], have been found to have remained relatively constant in recent decades.

The results presented in this paper are one more piece of evidence that the socioeconomic gradient in health is persistent over time and suggest that the gradient may even have been exacerbated in Germany since the early 2000s. The findings point to a need for effective strategies to improve health opportunities for socially disadvantaged people. Strategies addressing different policy fields and focussing on material and structural living conditions are especially promising for improving health equity, as people’s living conditions are not only directly relevant to health but can also have indirect health effects through influencing behavioural and psychosocial factors [47, 48].

## Limitations

There are some study limitations worth noting. The data on SES and chronic illness were based on self-reports, which may be subject to information bias. It has been argued that self-report indicators of chronic morbidity can underestimate the extent of socioeconomic inequalities in ill health because people from lower SES groups may have higher thresholds for perceiving themselves as ill [49–51]. There are, however, empirical data that do not support this hypothesis and suggest that self-reported responses to questions on chronic illness are not essentially biased by SES [52]. Concerning the national representativeness of the survey samples, it must be mentioned that the response rate decreased across the surveys. Nonetheless, the sample bias according to key sociodemographic characteristics increased only slightly between 2003 and 2009 and remained constant thereafter, as discussed in our previous articles based on these data [26, 27]. To minimise the impact of potential selection bias from differential non-response across the surveys, we adjusted year-specifically for non-response using weighting factors (see above). As the weighting procedure considers the age, sex, educational level, and regional distribution of the samples, the national representativeness of the samples is limited to these characteristics. The intervals between the health surveys used were not equal, which may have potentially biased the estimation of trends. To prevent such bias, the size of intervals was considered in the statistical models.

## Abbreviations

SES: socioeconomic status
GEDA: German Health Update
SII: slope index of inequality
RII: relative index of inequality
